# The lengths of trachea and main bronchus in Chinese Shanghai population

**DOI:** 10.1038/s41598-021-81744-0

**Published:** 2021-01-26

**Authors:** Xiahui Ge, Haidong Huang, Chong Bai, Xuejun Guo, Christoforos Kosmidis, Konstantinos Sapalidis, Sofia Baka, Kosmas Tsakiridis, Wolfgang Hohenforst-Schmidt, Lutz Freitag, Anastasios Vagionas, Konstantinos Drevelegas, Paul Zarogoulidis

**Affiliations:** 1 Department of Respiratory Medicine, Shanghai Seventh People’s Hospital Affiliated to Shanghai University of Traditional Chinese Medicine, Shanghai, China; 2 Department of Respiratory Medicine, Changhai Hospital of Second Military Medical University, Shanghai, 200433 China; 3 Department of Respiratory Medicine, Xinhua Hospital Affiliated To Shanghai Jiao Tong University School of Medicine, Shanghai, 200092 China; 4 3rd University General Hospital, “AHEPA” University Hospital, Thessaloníki, Greece; 5 Oncology Department, “Interbalkan” European Medical Center, Thessaloníki, Greece; 6 Thoracic Surgery Department, “Interbalkan” European Medical Center, Thessaloníki, Greece; 7 Sana Clinic Group Franken, Department of Cardiology/Pulmonology/Intensive Care/Nephrology, “Hof” Clinics, University of Erlangen, Hof, Germany; 8 Department of Pulmonology, University Hospital Zurich, Rämistrasse 100, 8091 Zurich, Switzerland; 9 Oncology Department, (NHS) Genaral Hospital of Kavala, Kavala, Greece; 10 Radiology Department, “Euromedica” Private Diagnostic Laboratory, Thessaloníki, Greece

**Keywords:** Anatomy, Signs and symptoms

## Abstract

The knowledge of airway length is the theoretical basis in the diagnosis and management of airway disease. The objective of this study is to measure the length of trachea and left and right main bronchus in Chinese Shanghai population. A total of 153 consecutive adult patients with minor pulmonary disease in Xinhua hospital were enrolled for bronchoscopy examination. Measurements were conducted on head and neck neutral position and height, weight and age for each patient were recorded either. Student t test and multiple linear regression was used to compare means between males and females and to analyze correlation among height, weight, sexual dimorphism and the lengths of the trachea and bronchus. The lengths of the trachea and left main bronchus are significantly different between male and female patients (*P* < 0.01), but not for the lengths of right main bronchus between man and woman. Multiple linear regression analysis showed that height but not sexual dimorphism and weight correlated with the lengths of the trachea and right main bronchus. The lengths of the trachea and left main bronchus are significantly longer in males than in females. Moreover, height but not sexual dimorphism and weight influenced the length of airway.

## Introduction

Airway stenosis represents an emerging problem and its etiologies are multiple. Besides malignant airway obstruction, more benign airway stricture draws our attention. In china, endobronchial tuberculosis is the most common cause of benign airway stricture, followed by post intubation, post tracheostomy and airway trauma. It is vital to get the knowledge of airway length since it forms the theoretical basis in the diagnosis and management of airway disease. Up to date, data of airway length mainly come from corpse and chest CT scan. Few study report the airway length by bronchoscopy, especially lengths of left and right main bronchus. In this study, we enrolled 153 adult patients and measured their airway by bronchoscopy and further analyzed the correlation among length of airway and patients’ height, weight in males and females.

## Material and methods

### Study population

Between January 2012 and March 2016, A total of 159 consecutive adult patients were initially enrolled at the bronchoscopy unit in Xinhua hospital,china. Patients with small pulmonary lesions in thoracic CT scan which do not alter airway dimension and position were registered. Six patients with lung cancer were excluded due to malignant central airway infiltration. Therefore, 153 adult patients were eligible for evaluation in the study, including 72 patients with unilateral mild pneumonia, 60 patients with pulmonary small nodule (major diameter ≤ 1 cm) and 21 patients with no lesion in chest CT scan but chronic cough or occasional bloody sputum. Among 153 patients, 45%(69/153) male patients and 55% (84/153) female patients were enrolled for bronchoscopy examination. A written informed consent was obtained from all patients. Not only the lengths of trachea and bronchus but age, height, and weight for each patient were recorded. This study was approved by the Ethics Committee of Xinhua Hospital Affiliated to Shanghai Jiaotong University School of Medicine.

### Bronchoscopy examination

After preparation including fasting prior to the procedure(> 6 h) and local anesthesia with 2% lidocaine by nebulization, bronchoscopy examination was transnasally performed by using a flexible bronchoscope (Olympus BF-260). Continous pulse oximetry, blood pressure, heart rate and an electrocardiogram were monitored during the whole procedure. All patients lied in a supine position and one nurse helped their head and neck on the neutral position(not flexion and extension position). After bronchoscopy examination, measurements of the lengths of the trachea and right and left main bronchus were performed.

### Measurements

In order to measure accurately the distance of the trachea and right and left main bronchus, two spring clips were used .When the distal end of the bronchoscopy reached the carina, one clip was marked on bronchoscopy at its point of nares. Then bronchoscopy was withdrawn at the point of vocal cord, another clip was used similarly to mark the nares position on bronchoscopy. The distance between two clip was determined, which was the tracheal length(distance from vocal cord to the carina). Similarly, the length of right main bronchi was to calculate the distance from right carina 1 to the carina and the length of left main bronchi was to measure the distance from left carina 2 to the carina. Measurements of the trachea and right and left main bronchi were repeated three times for each patient and was conducted by a single examiner (Xiahui Ge) in all patients. Since the trachea consists of cartilage and soft tissue, it is soft and elastic. When the head keep in extensive position, the airway will be stretched a little longer. On the contrary, the airway will be a little shorter when the head keep in flexion position. No matter the head kept in flexion or extension position, we haven’t found the tracheas be bent in any position.

The lengths of the trachea were associated with height. Both in female(r = 0.389, *P* < 0.01) and male (r = 0.707, *P* < 0.01)patients. There was no correlation between airway length and age.

### Statistical analysis

Lengths of the trachea and right and left main bronchus were recorded as mean and standard error. Student t test was used to compare means between male and female patients.To analyze correlation among height,weight, sexual dimorphism and the lengths of the trachea and right and left main bronchus, multiple linear regression was performed. All statistical tests were 2-side and *P* < 0.05 was considered statistically significant. Statistical software (SPSS for Windows, 23; SPSS, Chicago, IL) was used. URL: https://www.ibm.com/analytics/spss-statistics-software.

### Statement of human rights

 All procedures performed in studies involving human participants were in accordance with the ethical standards of the institutional and/or national research committee and with the 1964 Helsinki declaration and its later amendments or comparable ethical standards.

## Results

In our study, 69 male and 84 female patients were studied.The mean age was 53 years (range from 20 to 75) for male patients and 51 years (range from 18 to 75) for female patients and the mean weight and height was also shown in Table 1.

**Table 1:** Characteristics and lengths of trachea and bronchus in adult patients.

Variables	Male	Female	Combined
No. of patients	69	84	153
Age(year)	52.65 ± 13.82	50.82 ± 14.59	51.65 ± 14.23
Tracheal length (cm)	13.78 ± 1.10	12.9 ± 1.23	13.29 ± 1.25
Range	11.5–16.0	9.9–15.6	9.9–16.0
LMB length (cm)	4.71 ± 0.45	4.47 ± 0.47	4.58 ± 0.48
Range	4.0–5.8	3.5–5.7	3.5–5.8
RMB length (cm)	1.96 ± 0.62	1.75 ± 0.67	1.84 ± 0.65
Range	1.0–3.5	1.0–3.7	1.0–3.7
Height (cm)	170.55 ± 5.17	160.88 ± 6.09	165.24 ± 7.45
Weight (kg)	67.17 ± 10.73	57.38 ± 8.21	61.79 ± 10.60

*LMB* left main bronchus, *RMB* right main bronchus.

**Figure 1:**
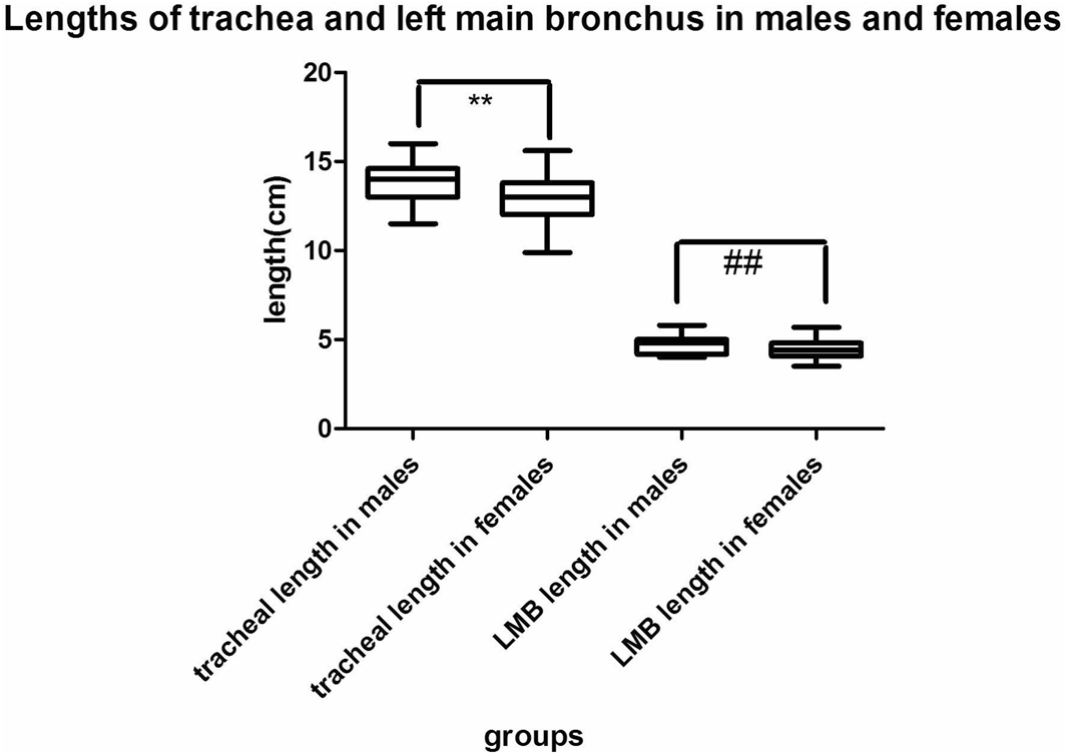
Lengths of trachea and left main bronchus in males and females. The lengths of the trachea and left main bronchus are significantly longer in males than those in females. (LMB left main bronchus;** represent the comparison the length of trachea between males and females.*, *P* < 0.05; **, *P* < 0.01. # represent length of left main bronchus compare to males and females. #, *P* < 0.05; ##, *P* < 0.01).

The lengths of the trachea and left and right main bronchus were 13.78 ± 1.10 cm, 4.71 ± 0.45 cm and 1.96 ± 0.62 cm in man and 12.9 ± 1.23 cm, 4.47 ± 0.47 cm,1.75 ± 0.67 cm in woman, respectively. The lengths of the trachea and left main bronchus are significantly different between male and female patients (*P* < 0.01), but no significant difference for the lengths of right main bronchus between men and women (*P* = 0.052) Fig. 1 and Table 2.

**Table 2:** Lengths of trachea and bronchus in males and females.

Variables	Gender	No. of patients	Length mean (SD)	t value	*P* value
Trachea	Males	69	13.78 ± 1.10	− 4.609	< 0.01
	Females	84	12.9 ± 1.23		
LMB	Males	69	4.71 ± 0.45	− 3.172	< 0.01
	Females	84	4.47 ± 0.47		
RMB	Males	69	1.96 ± 0.62	− 1.961	0.052
	Females	84	1.75 ± 0.67		

In our study, the lengths of the trachea and right main bronchus were associated with sexual dimorphism and height. However, height between men and women was significantly different. Therefore, multiple linear regression was applied to assess which factor influence the lengths of the trachea and right main bronchus. As shown in Table 2, height but not sexual dimorphism and weight showed significance correlation with the lengths of the trachea (Bata = 0.536, t = 7.803, *P* < 0.01)and right main bronchus(Bata = 0.0.238, t = 3.012, *P* < 0.01). However, sexual dimorphism correlate with the length of left main bronchus (Bata = 0.250, t = 3.172, *P* < 0.01).

## Discussion

With the improvement of airway disease’ cognition, more benign tracheobronchial stenosis diseases(i.e. post intubation, post tracheostomy,endobronchial tuberculosis) are paid great attention among pulmonologist, professional doctors in critical illness and anesthesiologist. Surgical resection and bronchoscopy intervention are the procedure of choice for most of the patients with benign airway strictures, which are not suitable for extensive airway stenosis. More methods have been developed to solve the problem. Successful implantation of cartilage autograft and transplantation of aortic allograft were reported in prior studies^1,2^. Moreover, recent study has used epithelial cells and mesenchymal stem cell-derived chondrocytes seeded onto tissue-engineered tracheal matrices synchronously and showed normal function and no signs of immunologic rejection in 30-year old woman with end-stage bronchomalacia^3^. Whatever tracheal tissue engineering or stent implantation is involved in repairing airway, the length and diameter of trachea and bronchus lays the foundation for the diagnosis and treatment of airway stenosis.

To determine the lengths of trachea and bronchus in normal people in south china, mild pulmonary diseases in Xinhua hospital were enrolled in the study, which did not influence airway dimension and position. With the application of computed tomography (CT) scanning for annual checkup in south china, more patients with pulmonary nodule are detected, especially pulmonary ground-glass opacity (GGO). Due to no alteration the location of airway, 39% (60/153) patients with pulmonary GGO (diameter range from 1 to 2 cm)were advised to accept bronchoscopic examination and were brought into our study.There were 21 patients with normal chest CT scan and pulmonary function test joining in our study, including 18 patients complaining of chronic cough and 3 patients with occasional bloody sputum. About half of the participants were patients with unilateral mild pneumonia. Therefore, determination of these participants represented the normal airway data in Chinese Shanghai population.

Prior studies indicated that head and neck movements partially influenced the length of airway. Head and neck flexion made the endotracheal tube(ETT) move toward the carina, while extension withdrew the tube toward the head^4,5^. Further study reported that not only flexion and extension but head rotation altered ETT position^6^. Therefore, each patients head, in our study, was positioned neutrally (not flexion and extension position) and one nurse ensured the patient’s head on neutral position during the whole procedure. Data collected from the study reflected the length of airway in vivo on neutral position.

In the past, data of the airway length and cross-section were collected from necropsy^7,8^. However, in different head and neck position^4–6^ and under different physiological conditions, the lengths of the trachea and bronchus varied slightly. Therefore, data from necropsy in previous studies could not reflect the true length in vivo. The measurements of tracheal length reported here were larger than those from prior cadaver reports. In the study of José et al.^9^, the distance from first tracheal ring to carina verge was determined to represent the tracheal length, in which subglottic larynx length was not included. Moreover, shorter results from autopsy were also due to shrinkage of the specimen. However, method to measure the dimension and length of airway in vivo was investigated either. In the early 1950s, chest radiographs was used to measure tracheal diameter^10^ and a large number of cases were subsequently measured in another study^11^. In recent years, CT scan was adopted, which provided not only dimension and length of airway but also interbronchial angle^12^ and airway area. Results of the present study showed that our data were also larger than those from chest radiographs and CT scan. Distance from the lower edge of the cricoid to the ridge of the carina defined as tracheal length might account for for the shorter results^13,14^.

The lengths of trachea in man and woman in the present study were 13.78 ± 1.10 cm and 12.9 ± 1.23 cm, which was consistent with previous study^5^. To date few studies determine the lengths of left and right main bronchus in vivo by bronchoscopy. Those data formed the theoretical basis for the operation and endotracheal intubation and stent placement. In the study of Hartrey et al.^5^, man had longer trachea than woman, but no significant difference was found due to a small number of cases.One recent study reported that trachea in male gender significant longer than that in female gender^14^. In agreement with previous study,the present study showed that lengths of trachea and left main bronchus in male gender were significantly longer than that in female gender, but no significant difference for the lengths of right main bronchus between men and women. Not large enough cases in our study might account for no significant difference for right main bronchus in males and females.

In our study, the lengths of the trachea and left main bronchus were associated with sexual dimorphism and height. As we all known, height between men and women was significantly different either. Whether sexual dimorphism exert direct or indirect effect on the lengths of the trachea and left main bronchus should be analyzed further. Multiple linear regression showed that height instead of sexual dimorphism and weight correlated with the lengths of the trachea and left main bronchus. So far, few study report the lengths of the trachea and left main bronchus correlate with height.

There are several limitations in our study. First, data were collected from the small number of cases and only from Xinhua hospital, which only represented length of airway in south china Shanghai population. Second, unblinded observers involving in the study might introduce a source of bias. Therefore, multicenter study in china should be carried out, age, sexual dimorphism, height and weight are need to be analyzed in the future.

In conclusion, the lengths of trachea and bronchus in our study were measured on head and neck neutral position by bronchoscopy in Chinese Shanghai population, which represented the length of airway in vivo on neutral position in south china. Moreover, lengths of trachea and left main bronchus in male gender were significantly longer than those in female gender, but no significant difference for the lengths of right main bronchus in males and females. By multiple linear regression analysis, height instead of sexual dimorphism correlated with the lengths of the trachea and left main bronchus. The measurements in our study form the theoretical basis for the operation and endotracheal intubation and bronchoscopy intervention in the future.

## Abbreviations

CT: Computed tomography
GGO: Ground-glass opacity
ETT: Endotracheal tube
