# Author Correction: The lengths of trachea and main bronchus in Chinese Shanghai population

**DOI:** 10.1038/s41598-021-98467-x

**Published:** 2021-10-01

**Authors:** Xiahui Ge, Haidong Huang, Chong Bai, Xuejun Guo, Christoforos Kosmidis, Konstantinos Sapalidis, Sofia Baka, Kosmas Tsakiridis, Wolfgang Hohenforst‑Schmidt, Lutz Freitag, Anastasios Vagionas, Konstantinos Drevelegas, Paul Zarogoulidis

**Affiliations:** 1 Department of Respiratory Medicine, Shanghai Seventh People’s Hospital Affiliated to Shanghai University of Traditional Chinese Medicine, Shanghai, China; 2 Department of Respiratory Medicine, Changhai Hospital of Second Military Medical University, Shanghai, 200433 China; 3 Department of Respiratory Medicine, Xinhua Hospital Affiliated to Shanghai Jiao Tong University School of Medicine, Shanghai, 200092 China; 4 3rd University General Hospital, “AHEPA” University Hospital, Thessaloníki, Greece; 5 Oncology Department, “Interbalkan” European Medical Center, Thessaloníki, Greece; 6 Thoracic Surgery Department, “Interbalkan” European Medical Center, Thessaloníki, Greece; 7 Sana Clinic Group Franken, Department of Cardiology/Pulmonology/Intensive Care/Nephrology, “Hof” Clinics, University of Erlangen, Hof, Germany; 8 Department of Pulmonology, University Hospital Zurich, Rämistrasse 100, 8091 Zurich, Switzerland; 9 Oncology Department, (NHS) Genaral Hospital of Kavala, Kavala, Greece; 10 Radiology Department, “Euromedica” Private Diagnostic Laboratory, Thessaloníki, Greece

Correction to: *Scientific Reports* 10.1038/s41598-021-81744-0, published online 26 January 2021

The original version of this Article contained errors in the Material and Methods section under subheading ‘Study population’,

“Therefore, 153 adult patients were eligible for evaluation in the study, including 71 patients with unilateral mild pneumonia, 60 patients with pulmonary small nodule (major diameter ≤ 1 cm) and 21 patients with no lesion in chest CT scan but chronic cough or occasional bloody sputum.”

now reads:

“Therefore, 153 adult patients were eligible for evaluation in the study, including 72 patients with unilateral mild pneumonia, 60 patients with pulmonary small nodule (major diameter ≤ 1 cm) and 21 patients with no lesion in chest CT scan but chronic cough or occasional bloody sputum.”

Furthermore, in the Material and Methods section under subheading ‘Measurements.’,

“Similarly, the length of right main bronchi was to calculate the distance from left carina 2 to the carina and the length of left main bronchi was to measure the distance from right carina 1 to the carina.”

now reads:

“Similarly, the length of right main bronchi was to calculate the distance from right carina 1 to the carina and the length of left main bronchi was to measure the distance from left carina 2 to the carina.”

Lastly, in the Results section,

“However, height between men and women was significantly different either.”

now reads:

“However, height between men and women was significantly different.”

The original Article has been corrected.

